# Curcumin- and capsaicin-loaded nanoemulsions improve oxidative stress, intestinal morphology, and feed efficiency in slow-growing Korat chickens under high stocking density

**DOI:** 10.1016/j.psj.2026.106513

**Published:** 2026-01-23

**Authors:** Wichuta Khosinklang, Pramin Kaewsatuan, Piyaradtana Homyok, Mutyarsih Oryza, Nitipol Chainet, Teerapong Yata, Naiyaphat Nittayasut, Pakpoom Boonchuen, Amonrat Molee, Tom E. Porter, Wittawat Molee

**Affiliations:** a School of Animal Technology and Innovation, Institute of Agricultural Technology, Suranaree University of Technology, Nakhon Ratchasima 30000, Thailand; b Premier Innova CO., LTD, 1 Premier Corporate Park, Soi Premier 2, Srinakarin Road, Nong Bon Subdistrict, Prawet District, Bangkok 10250, Thailand; c School of Biotechnology, Institute of Agricultural Technology, Suranaree University of Technology, Nakhon Ratchasima 30000, Thailand; d Department of Animal & Avian Sciences, University of Maryland, College Park, Maryland, USA

**Keywords:** Curcumin, Capsaicin, Nanoemulsion, Stocking density, Oxidative stress

## Abstract

This study investigated the effects of curcumin (**CUR**)- and capsaicin (**CAP**)- loaded nanoemulsion on blood biochemical changes, oxidative status, jejunal morphology, inflammatory parameters, and performance of slow-growing Korat chickens (**KRC**) raised under high stocking density (**HSD**). A total of 480 male KRC (21 d of age) were allocated into four groups: (1) HSD without supplementation, (2) normal stocking density (**NSD**) without supplementation, (3) HSD supplemented with CUR and CAP in powdered form (**P-CUR+CAP**), and (4) HSD supplemented with CUR- and CAP-loaded nanoemulsions (**NE-CUR+CAP**). Chickens receiving NE-CUR+CAP showed no adverse changes in liver or kidney function compared with other groups. The heterophil-to-lymphocyte ratio was reduced in NE-CUR+CAP group relative to HSD group and was comparable with NSD and P-CUR+CAP groups (P < 0.05). NE-CUR+CAP also lowered levels of TBA in the liver and jejunum while enhancing hepatic superoxide dismutase activity compared with HSD group (P < 0.05). Villus height, villus height-to-crypt depth ratio, anti-inflammatory response, and cecal *Lactobacillus* counts were improved, whereas crypt depth and cecal *Escherichia coli* were reduced in the NE-CUR+CAP group (P < 0.05). Although feed intake, BW, and body weight gain were not affected, the feed conversion ratio was significantly lower in NE-CUR+CAP compared with HSD group (P < 0.05). In conclusion, NE-CUR+CAP mitigated oxidative stress and inflammation, improved intestinal health, and enhanced feed efficiency in slow-growing chickens raised under HSD.

## Introduction

Slow-growing chickens **(SG)** provide tastier meat, higher collagen, and lower fat, and are considered an important source of protein (Katemala et al., 2021). They also contribute to economic well-being and food security in many nations (Jobe et al., 2019). These chickens are primarily raised by small- to medium-scale farmers, particularly in developing countries, who often face challenges in competing with larger enterprises due to limited competitiveness (Wilson et al., 2022). However, raising SG strains requires longer production cycles compared to commercial breeds, which can adversely affect household income (Bungsrisawat et al., 2025). Consequently, increasing the stocking density of chickens per unit area has been proposed as a strategy to improve productivity. However, high stocking density (**HSD**), a major source of stress, increases free radical production, which can damage biological molecules including cell membranes (Thiamhirunsopit et al., 2014). This stress negatively affects health, performance, and welfare, ultimately leading to economic losses for farmers. Therefore, there are concerns regarding potential compromises in both chicken welfare and productivity under HSD.

Over the past decade, various nutritional strategies including plant extract supplementation have been employed to alleviate oxidative stress and improve the performance of chickens raised at HSD. Examples include stem bark extract, licorice extract, curcumin (**CUR**) extract, *Forsythia suspensa* extract, and garlic powder (Sugiharto, 2022). Among phytogenic compounds, the combination of CUR and capsaicin (**CAP**) – the active ingredients of turmeric (*Curcuma longa*) and capsicum oleoresin derived from red pepper, respectively – show considerable promise. Both compounds possess pharmacological and therapeutic properties, including antioxidant, antimicrobial, and anti-inflammatory activities (Choi et al., 2011; Lagana et al., 2019). They also enhance nutrient digestibility and metabolism, promote beneficial gut microbiota, and support intestinal integrity (Reda et al., 2020). Previous studies demonstrated that combining turmeric and capsicum enhanced BW, reduced gut inflammation, and modulated immune responses in chickens (Lee et al., 2011, 2013). Given these benefits, CUR and CAP together may exert greater protective effects than single antioxidants in maintaining normal physiological functions in chickens under stress conditions.

However, the poor solubility and stability of CUR (Marwa et al., 2023), along with the extreme irritation caused by CAP (Han et al., 2020), result in low bioavailability, which remains a major challenge. In addition, conventional feed and feed additives are prone to degradation during storage and processing and lack controlled-release properties, leading to wastage, inefficient utilization, and reduced cost-effectiveness. Nanotechnology offers significant advantages for feed supplementation. Nano-based formulations enable targeted delivery to specific organs and biological barriers, improving oral bioavailability and absorption efficiency while reducing environmental impact through minimized waste (Nile et al., 2020). Previous studies have indicated that dietary supplementation with 200 to 400 mg/kg nanocurcumin improved oxidative status in poultry by increasing superoxide dismutase (**SOD**) and glutathione sulfhydryl (**GSH**) (Reda et al., 2020) and by decreasing malondialdehyde (**MDA**) (Partovi et al., 2020; Rahmani et al 2018) and **TBA** (Partovi et al., 2019). To date, no studies have investigated the combined use of CUR and CAP in nanoparticle form in chicken feed. Therefore, investigating the antioxidant properties of CUR- and CAP-loaded nanoemulsions is necessary for advancing poultry nutrition and production.

The objective of the present study was to examine the effects of CUR- and CAP-loaded nanoemulsions on blood biochemical parameters, oxidative status, jejunal morphology, microbial populations, inflammatory indices, and growth performance of SG raised under HSD. Korat chickens (**KRC**), a Thai crossbreed, were used as a SG chicken model. The results are expected to provide both fundamental insights into nano-antioxidant delivery and practical strategies to enhance animal welfare and economic viability in poultry production.

## Materials and methods

### Preparation of curcumin- and capsaicin-loaded nanoemulsions

The nanoemulsion was fabricated using elevated temperature and high homogenization energy. Initially, the oil phase was prepared by adding 20 mL of medium chain triglyceride **(MCT**, Dubois-Natural Esters, Sdn. Bd., Pasir Gudang, Malaysia**)** to a hotplate stirrer set at 80°C and 500 rpm. Subsequently, the aqueous phase mixture was prepared by mixing 80 mL of modified starch solution (SMS Corporation Co., Ltd., Pathum Thani, Thailand) with 1 g of CUR (purity ≥ 98%) or CAP (purity ≥ 95%) **(**Chanjao Longevity Co., Ltd., Bangkok, Thailand**),** as determined by high performance liquid chromatography (**HPLC**), using a hotplate stirrer. Following this, the hot aqueous phase mixture was carefully poured into the oil phase under mechanical stirring at 400 to 500 rpm for 15 min at 80°C. Once the aqueous and oil phases were thoroughly mixed, they were homogenized using a high-speed homogenizer (IKA Works, Inc., Germany) at 8000 rpm for 15 min, followed by sonication in a sonicator unit (Qsonica sonicators, USA) set at 30 Amp pulse on 30 s and off 10 sec intervals for 5 min. After that, the nano characteristics, such as particle size, polydispersity index, and zeta potential, were measured using a Zetasizer Nano ZS (Malvern Instruments, UK). All nanoemulsions were fed into a spray dryer (Model B-290, BÜCHI Labortechnik AG, Switzerland) to produce microcapsules. The inlet and outlet temperatures were maintained at 130°C and 70°C, respectively, with a feed flow rate of 10 mL/min.

### Ethics statement

All procedures in the present study were approved by the Ethics Committee on Animal Use of the Suranaree University of technology (**SUT**), Nakhon Ratchasima, Thailand (SUT-IACUC-0015/2022).

### Birds, experimental design, and diets

The experiment was conducted from April to July 2024 at the SUT farm. The temperatures ranged from 24.1–42.5°C, with an average RH of 65.1%. All chickens originated from the same hatch and were reared under identical management and environmental conditions at the SUT farm until 21 d of age.

At 21 d, a total of 480 male KRC with an average BW of 265.81 ± 35.19 g were randomly allocated to 4 treatments in a completely randomized design, with 6 replicates (pens) per treatment and 20 birds per pen. Chickens were housed in floor pens within an open-sided housing system. Before bird placement, the house and pens were thoroughly cleaned and sprayed with disinfectants. Each pen was bedded with approximately 5 cm of rice husk. Pen size was adjusted to achieve 2 stocking densities: 8 birds/m² (normal stocking density; **NSD**) using pens of 2.5 m², in accordance with the recommendation of the National Bureau of Agricultural Commodity and Food Standards (2017), and 16 birds/m² (high stocking density; HSD) using pens of 1.25 m². Natural lighting was provided after the brooding period. Ventilation was primarily natural through the open-sided structure, with electric fans used as needed to maintain adequate airflow. Plastic sheeting was installed around the house to reduce wind exposure throughout the experimental period.

All birds received the same basal diet (Table 1); however treatments differed in stocking density and dietary supplementation as follows: (1) HSD (16 birds/m²) without supplementation; (2) NSD (8 birds/m²) without supplementation; (3) HSD supplemented with 200 mg/kg CUR and 2 mg/kg CAP in powdered form (**P-CUR+CAP**); and (4) HSD supplemented with 200 mg/kg CUR and 2 mg/kg CAP in nanoemulsion form (**NE-CUR+CAP**). Birds in the P-CUR+CAP and NE-CUR+CAP treatments received identical inclusion levels of CUR and CAP. HPLC analysis confirmed the presence of CUR and CAP in the final diets. In the NE-CUR+CAP treatment, the measured concentrations were 173.52 ± 1.56 mg/kg for CUR (recovery: 86.76%) and 1.78 ± 0.25 mg/kg for CAP (recovery: 88.97%). In the P-CUR+CAP treatment, the corresponding concentrations were 177.93 ± 1.55 mg/kg for CUR (recovery: 88.97%) and 1.79 ± 0.11 mg/kg for CAP (recovery: 89.33%). These measured values were slightly lower than the calculated target levels.

**Table 1:** Ingredients and nutrient composition of the basal diets (g/100 g diet, as-fed basis).

Item	Grower (d 22 to 42)	Finisher (d 43 to 63)
Soybean meal	6.0	5.0
Corn	57.0	64.0
Full fat soybean	33.0	27.6
DL-Met	0.05	-
L-Lys.HCl	0.25	0.17
Salt	0.34	0.33
Calcium carbonate	1.56	1.35
Monocalcium phosphate (21% P)	1.30	1.05
Vitamin-Mineral premix 1	0.50	0.50
**Calculated nutrients (%)**		
ME (Kcal/kg)	3100	3100
CP	19.0	17.0
Digestible Lys	1.00	0.84
Digestible Met	0.43	0.35
Digestible Met + Cys	0.74	0.63
Digestible Thr	0.86	0.81
Calcium	0.95	0.81
Available phosphorus	0.47	0.40
**Analyzed composition (%)**		
Dry matter	91.4	91.1
CP	19.2	17.2

1 Vitamin-Mineral premix (0.5%) provided the following per kilogram of diet: 15,000 IU of vitamin A; 3000 IU of vitamin D3; 25 IU of vitamin E; 5 mg of vitamin K3; 2 mg of thiamine; 7 mg of riboflavin; 4 mg of pyridoxine; 25 µg of cobalamin; 11.04 mg of pantothenic acid; 35 mg of nicotinic acid; 1 mg of folic acid; 15 µg of biotin; 250 mg of choline chloride; 1.6 mg of copper; 60 mg of manganese; 45 mg of zinc; 80 mg of iron; 0.4 mg of iodine; 0.15 mg of selenium.

Experimental diets were formulated to be isonitrogenous and isocaloric, providing 3,100 kcal/kg ME and 19% CP during the grower phase (d 22 to 42) and 17% CP during the finisher phase (d 43 to 63). Feed and water were provided *ad libitum* throughout the experimental period. Feed was supplied twice daily at 07:00 and 16:00 h. Each pen was equipped with one feeder cup (40 cm diameter × 30 cm height) and three nipple water drinkers.

### Measurements and chemical analyses

#### Growth performance

Growth performance was evaluated weekly by measuring BW and feed intake (**FI**). Body weight gain (**BWG**) and feed conversion ratio (**FCR**) were then calculated. The mortality rate was also recorded.

#### Sample collection

At 63 d of age, blood samples were collected from 12 males per treatment (2 birds per replicate pen) via the jugular vein. Samples were placed into lithium heparin tubes for plasma biochemical and pro-inflammatory cytokine analyses and into EDTA tubes for hematological analysis. After that, 48 chickens (12 birds per treatment; 2 birds per replicate pen) underwent a 12-h feed withdrawal period and were then transported to the university slaughterhouse, where they were electrically stunned prior to slaughter. The jejunum was excised, cut into 2-cm segments and immediately fixed in 10% neutral buffered formalin for 24 to 48 h at room temperature for morphological analysis. The remaining jejunum tissue and liver samples were frozen at -80 °C for measurement of oxidative stress parameters. Cecal contents were collected from each bird by gentle squeezing into sterile tubes, and the samples were stored at -80 °C for microbial population enumeration.

#### Blood parameters

All blood parameter analyses were measured at a veterinary laboratory (Vet Central Lab, Nakhon Ratchasima, Thailand). Whole blood samples were analyzed for red blood cells (**RBC**), hemoglobin (**Hb**), packed cell volume (**PCV**), white blood cells (**WBC**), monocytes, basophils (**Baso**), eosinophils, heterophils (**H**), lymphocytes (**L**), and H/L ratio. Plasma samples were used to determine concentrations of alanine aminotransferase (**ALT**), aspartate aminotransferase (**AST**), creatinine, and blood urea nitrogen (**BUN**).

#### Pro-inflammatory cytokine measurements

Serum samples were used for cytokine analyses. Tumor necrosis factor alpha (**TNF-alpha**) and IL-6 concentrations were measured using ELISA kits (MyBioSource, China), while IL-2 was quantified using the Milliplex® Chicken Cytokine/Chemokine Premixed 12-plex Panel 1 (Millipore Sigma, Burlington, MA, USA) following to the manufacturers’ instructions.

#### Antioxidant parameter analysis

Lipid peroxidation was evaluated by determining TBA concentrations through the measurement of MDA in liver and jejunum samples (500 mg each), following the method of Leick et al. (2010). Absorbance was measured at 532 nm using a Thermo Scientific Multiskan GO microplate spectrophotometer (Thermo Fisher Scientific Oy., Vantaa, Finland). The activity of SOD was assessed in liver samples (∼100 mg) using a SOD Assay Kit-WST (Dojindo, Kumamoto, Japan). The samples were homogenized in sucrose buffer, centrifuged at 10,000 × g for 60 min at 4°C, and then incubated with the WST working solution. Absorbance was recorded at 450 nm. Total GSH levels were quantified using a method adapted from Mochida et al. (2022). Liver tissues were homogenized in 5% sulfosalicylic acid (**SSA**), centrifuged at 8,000 × g for 10 min, diluted with double-distilled water and then reacted sequentially with buffer, enzyme, and coenzyme working solutions according to the instructions of the Glutathione Quantification Kit (Dojindo, Kumamoto, Japan). Absorbance was measured at 405 nm using the same microplate spectrophotometer. The levels of SOD, TBARS, and GSH were normalized to total protein content, which was determined using the Bradford assay (Pierce, Rockford, IL, USA), and expressed per mg protein.

#### Jejunal histology

Histological processing was performed on the jejunum samples previously collected and fixed. Samples were dehydrated, embedded in paraffin wax, and sectioned at 4 μm thickness using a microtome. Sections were mounted on glass slides and stained with hematoxylin-eosin. Villus height (**VH**), crypt depth (**CD**), and the VH/CD ratio were captured using an optical microscope (Eclipse E600, Nikon Corp) at 4x magnification fitted with a camera (XC77E, Sony Corn). Two cross-sections per sample were analyzed using the ImageJ software (version 1.54g; National Institutes of Health, Bethesda, MD, USA); the mean value was used for statistical analysis.

#### Bacterial populations

Cecal bacterial populations were determined following the method of Wang et al. (2017) with slight modifications. Briefly, 1 g of cecal content sample was homogenized in 9 mL of sterile water, and serial dilutions were prepared. A volume of 100 μL from the 10⁻³ and 10⁻⁴ dilutions, determined as suitable densities for *Lactobacillus* and *Escherichia coli (****E. coli****)*, respectively, was plated. Diluted samples were spread onto de Man, Rogosa and Sharpe agar plates and incubated anaerobically at 37°C for 24 h to enumerate lactic acid bacteria, and onto MacConkey agar plates incubated aerobically at 37°C for 24 h to enumerate *E. coli*. Bacterial populations were expressed as the logarithm of CFU per gram of cecal content sample.

### Statistical analysis

Data for growth performance, blood parameters, TBA in liver and jejunum, SOD activity in liver, jejunal morphology, cecal bacterial populations, and pro-inflammatory cytokines were analyzed using ANOVA in a completely randomized design. Analyses were performed using the GLM procedure of the SPSS software (version 16.0; SPSS Inc., Chicago, IL, USA). Results are presented as means ± SEM. Treatment means were compared using Tukey's multiple comparison test, with differences considered significant at P < 0.05.

An orthogonal contrast was conducted to compare the HSD group, designated as the negative control, with the NSD group, designated as the positive control. Additionally, the Dunnett test was used to compare the HSD control group with the HSD groups receiving CUR and CAP.

## Results and discussion

### The effect of curcumin- and capsaicin-loaded nanoemulsions on hematological indices in slow-growing Korat chickens

HSD is a major stressor in poultry and is commonly associated with alterations in hematological profiles and stress-related responses (Chloupek et al., 2011). The effects of CUR- and CAP-loaded nanoemulsions on hematological parameters of KRC under HSD are presented in Table 2**.** No significant differences were observed among treatments for RBC, Hb, PVC, WBC, monocytes, or eosinophils. However, chickens raised under HSD without supplementation exhibited the highest percentage of Baso (3.50%), H (67.0%), and H/L ratio (2.59), along with the lowest percentage of L (26.4%), compared with other treatments (P < 0.001). Notably, these parameters in the NE-CUR+CAP group were not different from those in the NSD group.

**Table 2:** Effect of curcumin- and capsaicin- loaded nanoemulsions on hematological and serum indices in slow-growing Korat chickens.

Items	Treatments				SEM	P-value	
	HSD	NSD	P-CUR+CAP	NE-CUR+CAP		Treatment	C^1^
Hematological indices							
RBC (x10^6^/mm^3^)	2.62	2.57	2.56	2.53	0.919	0.525	0.618
Hb (g/dL)	10.4	10.1	9.86	9.86	0.308	0.097	0.325
PVC (%)	31.2	30.3	29.7	29.7	0.934	0.092	0.329
WBC (cells/mm^3^)	7,163	7,590	7,026	7,025	325	0.950	0.667
Monocytes (%)	2.00	2.25	2.08	1.83	0.236	0.812	0.518
Basophils (%)	3.50^a^	1.43^c^	2.60^b*^	1.66^c*^	0.202	<0.001	<0.01
Eosinophil (%)	1.33	1.58	0.83	1.25	0.260	0.273	0.511
Heterophils (%)	67.0^a^	49.8^b^	59.2^ab^	52.7^b*^	1.764	<0.001	<0.01
Lymphocytes (%)	26.4^b^	44.2^a^	36.7^a*^	43.0^a*^	1.769	<0.001	<0.01
H/L ratio	2.59^a^	1.21^b^	1.65^b*^	1.29^b*^	0.128	<0.001	<0.01
Serum indices							
ALT (U/L)	7.40	7.14	7.00	7.10	0.707	0.966	0.784
AST (U/L)	208	210	208	208	7.911	0.994	0.840
Creatinine (mg/dL)	0.35	0.42	0.39	0.38	0.027	0.249	0.079
Blood urea nitrogen (mg/dL)	1.08	1.18	1.18	1.00	0.162	0.642	0.547

^a,b,c^ The different letters indicate significant differences (P < 0.05).

*Statistical differences (Dunnett test to compare the HSD with the HSD groups receiving P-CUR+CAP and NE-CUR+CAP).

^1^Contrast = HSD vs NSD.

HSD = chickens raised under high stocking density (16 birds/m^2^) without supplementation;

NSD = chickens raised under normal stocking density (8 birds/m^2^) without supplementation;

P-CUR+CAP = chickens raised under HSD with received curcumin and capsaicin in the powder form; NE-CUR+CAP = chickens raised under HSD with received curcumin- and capsaicin-loaded nanoemulsions.

ALT= alanine amino transferase; AST= aspartate amino transferase.

These results support the hypothesis that HSD induces oxidative stress, reflected by an elevated H/L ratio, a well-established indicator of stress in poultry. Supplementation with CUR and CAP effectively mitigated these stress responses, consistent with previous studies (Meena et al., 2022; Nasr et al., 2021). Altan et al. (2000) similarly reported increased Baso percentage in chickens exposed to 39°C, which is an indicator of stress responses (Sokolenko et al., 2024). This response is likely mediated by stress-induced activation of the hypothalamic-pituitary-adrenal axis, leading to elevated corticosterone levels (Timmermans et al., 2019). Elevated corticosterone suppresses L proliferation in lymphoid organs while promoting H release, resulting in an increased H/L ratio (Hangalapura et al., 2004; Trout et al., 1995).

Furthermore, chickens raised under NSD and those raised under HSD supplemented with CUR and CAP exhibited lower Baso percentage and H/L ratio, along with higher L percentage, compared with the unsupplemented HSD group (P < 0.001). These results align with previous studies reporting increased L numbers and reduced H/L ratio following supplementation with CUR or CUR nanoparticles (Rahmani et al., 2018), CAP (Prieto and Campo, 2010), or combinations of turmeric and cayenne pepper (Adegoke et al., 2018). Collectively, these findings indicate that dietary CUR and CAP enhance antioxidant capacity and alleviate stress responses in chickens raised under HSD, resulting in hematological profiles comparable to those under NSD.

### The effect of curcumin- and capsaicin-loaded nanoemulsions on serum indices in slow-growing Korat chickens

In this study, NE-CUR+CAP was developed to enhance the bioavailability of CUR and CAP in chickens. Serum ALT and AST were measured as indicators of liver function, whereas BUN and creatinine were measured as indicators of kidney function (Valchev et al., 2014). Neither powder nor nanoemulsion forms of CUR and CAP significantly affected these serum indices (Table 2). Consistent with our hypothesis, chickens receiving NE-CUR+CAP showed no adverse effects, indicating that the nanoemulsion is safe for hepatic and renal function. This safety may be attributed to the nanoemulsion components, including medium-chain triglycerides and modified starch, which are generally recognized as safe (**GRAS**) (Marten et al., 2006; Zhang et al., 2020).

Conversely, previous studies have reported beneficial effects of CUR and CAP on serum liver enzyme activities. Hosseini-Vashan et al. (2012) demonstrated that heat-stressed chickens supplemented with 8 g/kg turmeric powder exhibited reduced ALP, AST, ALT, and LDH levels. Similarly, Badran et al. (2020) reported decreased AST activity in birds fed 25 or 100 mg/kg CUR and 50 or 100 mg/kg nano-CUR, while Adegoke et al. (2018) observed reduced ALT levels following supplementation with 400 g turmeric powder and 100 g *Capsicum frutescens* powder. These effects are attributed to the antioxidant and anti-inflammatory properties of CUR and CAP, including scavenging reactive oxygen species (**ROS**), enhancing endogenous antioxidant defenses, and inhibiting Nuclear Factor kappa B (**NF-κB**)-mediated inflammatory pathways, thereby protecting hepatocytes from oxidative damage and reducing hepatocellular injury (Adetunji et al., 2022; Manjunatha and Srinivasan, 2006; Relja et al., 2012; Zhang et al., 2024).

### The effect of curcumin- and capsaicin-loaded nanoemulsions on oxidation status in the liver and jejunum in slow-growing Korat chickens

**Fig. 1:**
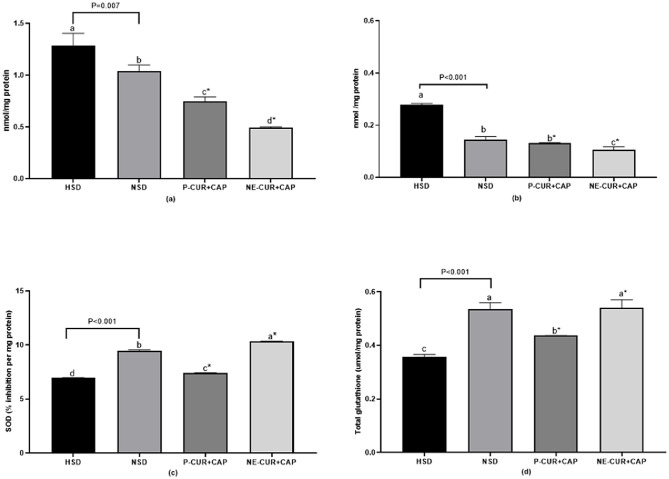
Effect of curcumin-and capsaicin-load nanoemulsion on TBARS in liver (a), and jejunum (b), SOD (c), and total glutathione activity (d) in the liver of slow-growing Korat chickens. ^a, b, c, d^ The different letters indicate significant differences (P < 0.05). *Statistical differences (Dunnett test to compare the HSD with the HSD groups receiving P-CUR+CAP and NE-CUR+CAP). P value indicated an orthogonal contrast between HSD and NSD groups. HSD = Chickens raised under high stocking density (16 birds/m^2^) without supplementation; NSD = chickens raised under normal stocking density (8 birds/m^2^) without supplementation; P-CUR+CAP = chickens raised under HSD with received curcumin and capsaicin in the powder form; NE-CUR+CAP = chickens raised under HSD with received curcumin-and capsaicin-loaded nanoemulsions.

The liver is a major metabolic organ with essential physiological functions (Chen et al., 2022), while the jejunum is highly susceptible to oxidative stress, which can induce inflammation, impair intestinal integrity, alter microbial balance, and negatively affect growth performance in chickens (Li et al., 2024). Chickens raised under HSD without supplementation exhibited the lowest SOD and GSH activities in the liver and the highest TBA levels in both the liver and jejunum compared with other treatments (P < 0.05; Fig. 1). In contrast, NE-CUR+CAP supplementation resulted in the highest liver SOD activity and the lowest TBA levels in both tissues (P < 0.05), while GSH levels were comparable to those observed in the NSD group.

These findings are consistent with previous studies reported that CUR and CAP enhance antioxidant status by upregulating antioxidant-related genes (Adetunji et al., 2022; Alizadeh and Kheirouri, 2019). The phenolic hydroxyl groups of CUR and CAP enable scavenging of hydroxyl radicals, superoxide ions, and nitric oxide through hydrogen atom transfer (De Lourdes Reyes-Escogido et al., 2011; Feng and Liu, 2009), while also chelating metal ions and inhibit metal ion-dependent free radical formation (Dai et al., 2010). Notably, the nanoemulsion form exhibited superior antioxidant efficacy compared with the powdered form, as indicated by enhanced SOD and GSH activities. Similar findings were reported by Rahmani et al. (2018), who showed that nanocurcumin reduced MDA levels more effectively than powdered CUR, and by da Silva et al. (2023), who observed greater free radical scavenging capacity of essential oil nanoemulsions. Abdel-Moneim et al. (2025) further reported dose-dependent increases in SOD activity and reductions in MDA following CUR nanoparticle supplementation. These enhanced effects are likely attributable to the nano-sized droplets and increased surface area of nanoemulsions, which improve interactions with free radicals.

Interestingly, chickens raised under NSD exhibited higher TBA levels in the liver and jejunum, and lower liver SOD activity than those receiving nanoemulsions (P < 0.05). This response may be explained by environmental conditions, as chickens can experience heat stress at temperatures as low as 18 to 24°C (Oke et al., 2024). In the present study, birds were raised in open housing where maximum temperature reached 42.5°C with an average humidity of 65.1%, suggesting that NSD birds were also exposed to heat stress. In contrast, HSD chickens supplemented with NE-CUR+CAP showed improved antioxidant status, highlighting the protective role of nanoemulsified CUR and CAP under heat stress conditions. Overall, these findings suggest that CUR- and CAP-loaded nanoemulsions effectively mitigate ROS, thereby reducing oxidative stress in chickens raised under HSD.

### The effect of curcumin- and capsaicin-loaded nanoemulsions on jejunum morphology in slow-growing Korat chickens

**Fig. 2:**
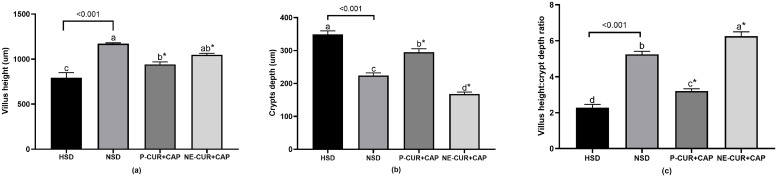
Effect of curcumin and capsaicin-load nanoemulsion on villus high (a), crypts depth (b), and ratio of villus high and crypts depth (c) of slow-growing Korat chickens. ^a, b, c, d^ The different letters indicate significant differences (P < 0.05). *Statistical differences (Dunnett test to compare the HSD with the HSD groups receiving P-CUR+CAP and NE-CUR+CAP). P value indicated an orthogonal contrast between HSD and NSD groups. HSD = Chickens raised under high stocking density (16 birds/m^2^) without supplementation; NSD = chickens raised under normal stocking density (8 birds/m^2^) without supplementation; P-CUR+CAP = chickens raised under HSD with received curcumin and capsaicin in the powder form; NE-CUR+CAP = chickens raised under HSD with received curcumin-and capsaicin-loaded nanoemulsions.

Oxidative stress can impair intestinal morphology by reducing epithelial cell proliferation, increasing CD, and decreasing VH (Mishra and Jha, 2019). Jejunal morphology and calculated indices are presented in **Fig. S1** and Fig. 2, respectively. Chickens raised under HSD without supplementation exhibited the lowest VH and VH/CD ratio and the highest CD compared with other treatments (P < 0.05). The VH/CD ratio is a critical indicator of nutrient digestion and absorption (Prakatur et al., 2019). Deeper crypts generally indicate rapid intestinal tissue renewal, often triggered by inflammation, tissue damage, or toxins produced by pathogens (Paiva et al., 2014). Accordingly, CD and VH/CD deserve particular attention.

Chickens receiving NE-CUR+CAP exhibited significantly lower CD and a higher VH/CD of jejunal epithelium compared with other groups (P < 0.05; Fig. 2**b** and Fig. 2**c**). These results are consistent with those of Fathi et al. (2024), who reported decreased CD and an increased VH/CD in chickens fed 100 to 200 mg/kg CUR nanoparticles. CUR may enhance intestinal epithelial cell turnover through the activation of cell mitosis, thereby increasing VH/CD (Samanya and Yamauchi, 2002). Similarly, Zhang et al. (2025) showed that 240 and 360 mg/kg CAP supplementation reduced CD and increased VH/CD in the jejunum of laying hens. These effects are likely mediated by the antioxidant and anti-inflammatory properties of CUR and CAP, which may reduce intestinal oxidative damage and preserve mucosal integrity (Xu et al., 2024; Zhang et al., 2025), as supported by reduced TBA level in the jejunum (Fig. 1**b**).

Interestingly, chickens recieving nanoemulsified CUR and CAP exhibited lower CD and higher VH/CD than those fed the powdered form (P < 0.05). Although VH did not differ significantly between forms, nanoemulsion-fed birds tended to show higher VH. The superior effects of NE-CUR+CAP may be attributed to the small droplet size and enhanced dissolution of hydrophobic compounds, which improve tissue penetration and bioavailability (Rashidi, 2021; Taghipour et al., 2018). In support, Ma et al. (2007) reported markedly greater bioavailability and tissue retention of nanoparticle-formulated CUR compared with native CUR in a rat model, whereas powdered CUR and CAP are rapidly metabolized and eliminated (Lopresti, 2018). Their limited absorption across the mucus layer and intestinal epithelium likely reduces efficacy under oxidative stress (Anand et al., 2007; Zheng and McClements, 2020). Overall, our findings suggest that the nanoemulsified CUR and CAP are more effective than powdered form in improving jejunal morphology and gut health in chickens.

### The effect of curcumin- and capsaicin-loaded nanoemulsions on bacterial populations in slow-growing Korat chickens

The cecum is a key site for microbial fermentation and plays a critical role in maintaining gut health. This study investigated the effects of CUR- and CAP-loaded nanoemulsions on cecal populations of *E. coli* and *Lactobacillus* in KRC (Table 3). Chickens raised under HSD without supplementation exhibited significantly lower cecal *Lactobacillus* counts and higher *E. coli* counts compared with other treatments (P < 0.001). Rearing birds under HSD in tropical environments is known to elevate ambient temperature due to limited air circulation, thereby inducing heat and oxidative stress (Wasti et al., 2020).

**Table 3:** Effect of curcumin- and capsaicin- loaded nanoemulsions on the growth of *E. coli* and *Lactobacillus* in the cecum of slow-growing Korat chickens.

Items (logCFU/g)	Treatments				SEM	P-value	
	HSD	NSD	P-CUR+CAP	NE-CUR+CAP		Treatment	C^1^
*Lactobacillus*	4.08^b^	4.38^a^	4.46^a*^	4.49^a*^	0.047	<0.001	0.018
*E. coli*	4.26^a^	4.13^b^	4.09^b*^	4.06^b*^	0.024	<0.001	0.004

^a,b^The different letters indicate significant differences (P < 0.05).

*Statistical differences (Dunnett test to compare the HSD with the HSD groups receiving P-CUR+CAP and NE-CUR+CAP).

^1^Contrast = HSD vs NSD.

HSD = chickens raised under high stocking density (16 birds/m^2^) without supplementation;

NSD = chickens raised under normal stocking density (8 birds/m^2^) without supplementation;

P-CUR+CAP = chickens raised under HSD with received curcumin and capsaicin in the powder form; NE-CUR+CAP = chickens raised under HSD with received curcumin- and capsaicin-loaded nanoemulsions.

In the present study, chickens in the NE-CUR+CAP, P-CUR+CAP, and NSD group showed increased *Lactobacillus* counts and reduced *E. coli* counts relative to the unsupplemented HSD group (P < 0.05). These findings align with Zhang et al. (2013), who reported that HSD promoted *E. coli* proliferation in the cecum. Elevated *E. coli* populations are associated with intestinal inflammation and colibacillosis, which may increase mortality in chicken flocks (Ncho, 2025; Tang et al., 2021).

Interestingly, powdered and nanoemulsified CUR and CAP produced comparable effects on cecal microbial populations, despite differences in absorption pathways. This finding contrasts with Rahmani et al. (2018), who reported that nanocurcumin supplementation increased *Lactobacillus* counts and reduced *E. coli* counts compared with birds fed diet a containing curcumin. One possible explanation is that only 5-10% of powdered CUR and CAP is absorbed in the small intestine, allowing larger quantities of unmetabolized polyphenols to reach the cecum. These compounds can be converted into bioactive metabolites that act as prebiotics, modulating microbial communities (Beslo et al., 2023). Conversely, the nanoemulsion form is more efficiently hydrolyzed and absorbed in the intestine as free fatty acids and monoacylglycerols, which are absorbed via passive diffusion and carrier-mediated transport in the intestine (Garaiova et al., 2007). Although this suggests less direct delivery to the cecum, we speculate that metabolites of the absorbed nanoform still influence the cecal environment, resulting in effects comparable to the powdered form.

In addition to direct microbial modulation, nanoemulsified CUR and CAP may indirectly improve gut microbiota through their antioxidant and anti-inflammatory activities. For instance, Li et al. (2023) demonstrated that CAP enhances intestinal antioxidant capacity through TRPV1/PKA/UCP and Keap1/Nrf2 pathways, while suppressing inflammation. A healthier intestinal barrier prevents harmful bacterial translocation and promotes colonization by commensal microbes (Di Vincenzo et al., 2024).

Collectively, these findings suggest that NE-CUR+CAP supplementation supports gut health in KRC raised under HSD by reducing pathogenic bacteria and promoting beneficial microbial populations.

### The effect of curcumin- and capsaicin-loaded nanoemulsions on inflammation biomarker levels (TNF-α, IL-2, and IL-6) in slow-growing Korat chickens

**Fig. 3:**
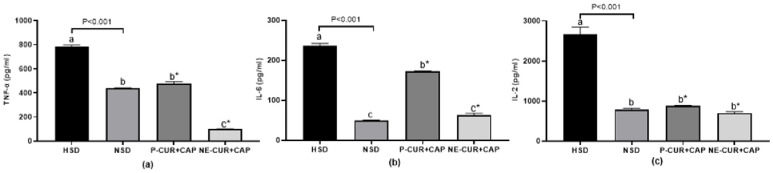
Effect of curcumin- and capsaicin-loaded nanoemulsions on serum inflammation biomarker levels including TNF-α (a), IL-6 (b) and IL-2 (c) in slow-growing chickens. ^a, b, c^ The different letters indicate significant differences (P < 0.05). *Statistical differences (Dunnett test to compare the HSD with the HSD groups receiving P-CUR+CAP and NE-CUR+CAP). P value indicated an orthogonal contrast between HSD and NSD groups. HSD = Chickens raised under high stocking density (16 birds/m^2^) without supplementation; NSD = chickens raised under normal stocking density (8 birds/m^2^) without supplementation; P-CUR+CAP = chickens raised under HSD with received curcumin and capsaicin in the powder form; NE-CUR+CAP = chickens raised under HSD with received curcumin-and capsaicin-loaded nanoemulsions.

Heat or oxidative stress can impair the intestinal mucosal barrier in chickens by increasing ROS production, which promotes pathogenic bacteria growth and elevates endotoxin concentrations. Absorption of these endotoxins stimulates pro-inflammatory cytokine production, leading to systemic inflammation (Abdel-Moneim et al., 2025; Hosseindoust et al., 2022). As shown in Fig. 3, chickens raised under HSD without supplementation showed the highest serum concentrations of TNF-α, IL-6, and IL-2 (P < 0.05). These results are consistent with Sakr et al. (2025), who reported upregulation of inflammatory markers (IL-6, IL-8, and IL-13) in chickens exposed to HSD conditions (22 birds/m²). Elevated cytokine levels under HSD likely result from stress-induced oxidative damage and immune dysregulation, which compromise intestinal health.

In contrast, chickens supplemented with CUR and CAP showed significantly reduced concentrations of pro-inflammatory cytokines compared with the unsupplemented HSD group (P < 0.05). These findings align with previous studies demonstrating that phytobiotic supplementation can reduce cytokine levels in poultry (Chodkowska et al., 2022; Eleiwa et al., 2023; Xu et al. 2024). Similarly, Hafez et al. (2022) demonstrated that dietary CUR (100 to 200 mg/kg) reduced TNF-α, IL-6 and IL-2 levels in broilers raised under HSD. Mechanistically, CUR exerts anti-inflammation by modulating NF-kB signaling (Zhang et al., 2024), while CAP reduces intestinal inflammatory through PPARγ activation (Mendivil., 2019). Together, these compounds regulate downstream cytokines including TNF-α, IL-1β, IL-2, IL-6.

Interestingly, cytokine concentrations in the NSD group were comparable to those observed in CUR- and CAP-supplemented HSD groups, indicating that supplementation restored inflammatory status toward normal conditions. Notably, the nanoemulsion form further reduced TNF-α levels compared with the NSD group (P < 0.05). This effect may be attributed to the combined anti-inflamatory and antioxidant actions of CUR and CAP. Nanoemulsified turmeric has been shown to enhance antioxidant defenses, including SOD and GSH activities (El-Sayed, 2024), which reduce ROS-mediated activation of NF-κB and subsequent cytokine expression (Morgan and Liu, 2010). This interpretation is supported by the improved antioxidant indices observed in the present study (Fig. 1**a-c**).

Moreover, the nanoemulsion form was more effective than the powder form in reducing TNF-α and IL-6 levels (P < 0.05), likely due to enhanced solubility, cellular uptake, and bioavailability (Abdel-Moneim et al. 2025; Geevarghese et al., 2023). Overall, these results indicate that CUR and CAP effectively alleviate systematic inflammation in chickens under HSD, with nanoemulsion delivery providing superior modulation of inflammatory responses through both direct and indirect mechanisms.

### The effect of curcumin- and capsaicin-loaded nanoemulsions on slow-growing Korat chicken growth performance

Raising chickens under HSD in tropical regions elevates oxidative stress (Nasr et al., 2021), leading to reduced FI, nutrient utilization, and growth performance (Najafi et al., 2015).

As expected, chickens in the unsupplemented HSD group exhibited significantly lower BW (P = 0.009) and BWG (P = 0.007) compared with those raised under NSD (Table 4). These findings are consistent with previous studies showing that HSD reduced BW due to elevated stress level (Nasr et al., 2021; Son et al., 2022), as further supported by the increased H/L ratio observed in the present study (Table 2). In contrast, chickens raised under HSD supplemented with CUR+CAP showed BW and BWG comparable to those in the NSD group. Similarly, Hafez et al. (2022) reported that broilers supplemented with 200 mg/kg CUR under HSD (20 birds/m²) exhibited BW comparable to birds raised at lower density (10 birds/m²). Chili supplementation has also been reported to improve BW under HSD, although responses vary depending on breed, plant source, and extract formulation (Thiamhirunsopit et al., 2014).

**Table 4:** Effect of curcumin- and capsaicin-loaded nanoemulsions on growth performance in slow-growing Korat chickens at 3 to 9 wk.

Items	Treatments				SEM	P-value	
	HSD	NSD	P-CUR+CAP	NE-CUR+CAP		Treatment	C^1^
Body weight (g)	1,112^b^	1,176^a^	1,149^ab^	1,161^ab^	8.813	0.048	0.009
Body weight gain (g)	854^b^	915^a^	888^ab^	901^ab^	8.503	0.049	0.007
Feed intake (g)	2,369^ab^	2,499^a^	2,322^b^	2311^b^	0.029	0.020	0.549
FCR	2.77^a^	2.73^ab^	2.61^ab^	2.57^b*^	26.14	0.027	0.133
Mortality rate (%)	2.50	0.83	1.67	0.83	0.44	0.575	0.238

^a,b^ The different letters indicate significant differences (P < 0.05).

*Statistical differences (Dunnett test to compare the HSD with the HSD groups receiving P-CUR+CAP and NE-CUR+CAP).

^1^Contrast = HSD vs NSD.

HSD = chickens raised under high stocking density (16 birds/m^2^) without supplementation;

NSD = chickens raised under normal stocking density (8 birds/m^2^) without supplementation;

P-CUR+CAP = chickens raised under HSD with received curcumin and capsaicin in the powder form; NE-CUR+CAP = chickens raised under HSD with received curcumin- and capsaicin-loaded nanoemulsions.

Regarding FI, chickens under HSD with CUR+CAP supplementation consumed less feed than those under NSD (P < 0.05). FI generally decreases with increasing stocking density (Miao et al., 2021; Tong et al., 2012), as birds raised under NSD (8 birds/m²) expend more energy on activity and mobility, requiring higher FI (Fanatico et al., 2007). In contrast, birds under HSD primarily consume feed to maintain homeostasis and cope with oxidative stress rather than to support growth (Oke et al., 2024), which may explain the absence of marked differences in FI among treatments.

Feed efficiency was significantly improved in chickens supplemented with NE-CUR+CAP compared with the unsupplemented HSD group (P < 0.05). Moreover, the FCR in birds receiving CUR+CAP in either powder or nanoemulsion form was comparable to that of the NSD group. The superior efficiency observed with the nanoemulsion may be related to its small particle size, which facilitates transcellular absorption (Mok et al., 2024). Furthermore, CUR and CAP are known to stimulate digestive enzyme secretion and enhance intestinal villus development, thereby improving nutrient utilization (Li et al., 2022; Rajput et al., 2013).

Overall, these findings indicate that CUR and CAP supplementation, particularly in nanoemulsion form, enhances feed efficiency and growth performance by improving nutrient digestibility and intestinal morphology, thereby alleviating the detrimental effects of HSD in slow-growing KRC.

## Conclusion

Dietary supplementation with CUR- and CAP-loaded nanoemulsions effectively alleviated the adverse effects of HSD in slow-growing chickens. Supplementation did not compromise liver or kidney function and significantly enhanced antioxidant capacity, gut morphology, cecal microbiota balance, inflammation status, and feed efficiency compared with unsupplemented birds raised under HSD conditions. In addition, CUR- and CAP-loaded nanoemulsions attenuated stress responses to levels comparable with those observed under NSD. Collectively, these findings highlight the potential of CUR- and CAP-loaded nanoemulsions as a targeted nutritional strategy to alleviate stress and performance impairments associated with HSD in poultry nutrition.

## CRediT authorship contribution statement

**Wichuta Khosinklang:** Writing – original draft, Visualization, Validation, Software, Resources, Methodology, Investigation, Formal analysis, Data curation, Conceptualization. **Pramin Kaewsatuan:** Writing – review & editing. **Piyaradtana Homyok:** Writing – review & editing, Investigation. **Mutyarsih Oryza:** Visualization, Investigation. **Nitipol Chainet:** Investigation. **Teerapong Yata:** Resources, Methodology. **Naiyaphat Nittayasut:** Resources, Methodology. **Pakpoom Boonchuen:** Writing – review & editing, Validation, Supervision, Methodology, Conceptualization. **Amonrat Molee:** Writing – review & editing, Supervision, Conceptualization. **Tom E. Porter:** Writing – review & editing, Supervision. **Wittawat Molee:** Writing – review & editing, Validation, Supervision, Resources, Project administration, Methodology, Funding acquisition, Conceptualization.

## Disclosures

The authors declare no conflicts of interest.

## Appendix Supplementary materials

Image, application 1
