# Supplementary material for: Curcumin- and capsaicin-loaded nanoemulsions improve oxidative stress, intestinal morphology, and feed efficiency in slow-growing Korat chickens under high stocking density
Source: Poult Sci. 2026 Jan 23;105(4):106513. doi: 10.1016/j.psj.2026.106513 (PMC12882722; doi:10.1016/j.psj.2026.106513)
Supplement: Supplementary file 1 [file mmc1.docx]

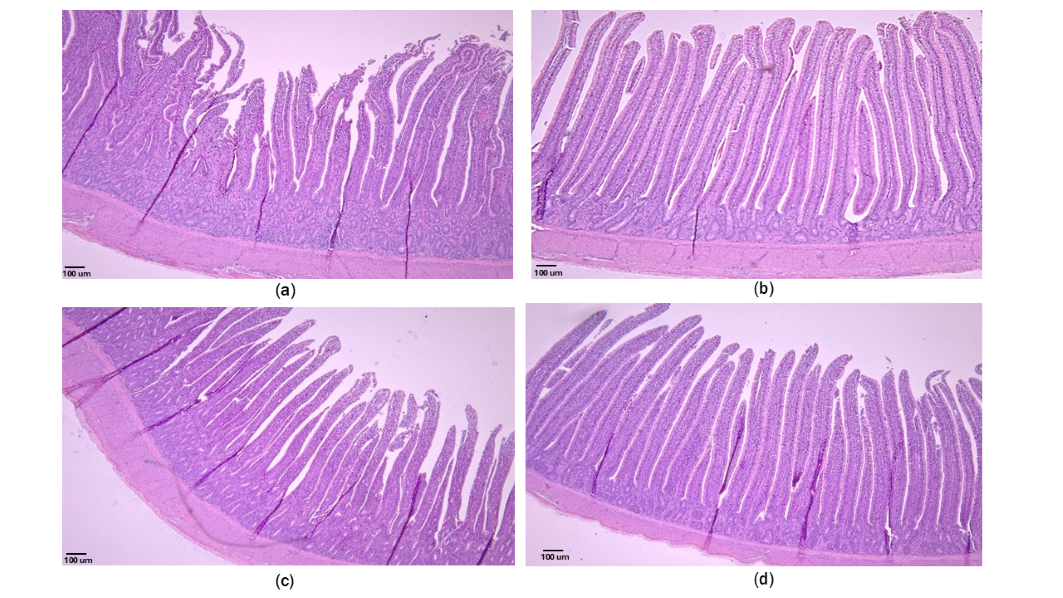


**Figure S1.** Jejunal morphology of chickens raised under high stocking density (HSD: 16 birds/m^2^) (a); chickens raised under normal stocking density (NSD: 8 birds/m^2^) (b); chickens raised under HSD with received curcumin and capsaicin in the powder form (P-CUR+CAP) (c); chickens raised under HSD with received curcumin-and capsaicin-loaded nanoemulsion (NE-CUR+CAP) (d).
